# Aortic Stenosis in End-Stage Renal Disease: Incidence, Prevalence, and Mortality in a National Korean Cohort

**DOI:** 10.3390/jcm14196921

**Published:** 2025-09-30

**Authors:** Minjeong Kim, Min-Ho Kim, Sang Jun Park, Hyangkyoung Kim

**Affiliations:** 1 Department of Cardiology, Ewha Womans University Medical Center, Ewha Womans University College of Medicine, Seoul 07985, Republic of Korea; 2 Department of Medicine, Graduate School, Yonsei University College of Medicine, Seoul 03722, Republic of Korea; 3 Ewha Medical Data Organization, Ewha Womans University Medical Center, Seoul 07804, Republic of Korea; 4 Department of Surgery, Ulsan University Hospital, University of Ulsan College of Medicine, Dong-gu, Ulsan 44033, Republic of Korea; 5 Department of Surgery, Ewha Womans University Medical Center, Ewha Womans University College of Medicine, Seoul 07985, Republic of Korea

**Keywords:** aortic valve stenosis, renal dialysis, kidney failure, chronic, mortality, cohort studies

## Abstract

**Background/Objectives**: Aortic stenosis (AS) is an increasingly recognized end-stage renal disease (ESRD) complication. This study aimed to identify AS incidence and prevalence in Korean patients with ESRD, assess the effect of AS on all-cause mortality, and determine associated risk factors unique to this population. **Methods**: This retrospective study used Korean National Health Insurance Service data from 2009 to 2021 and included adult patients with ESRD undergoing maintenance dialysis. AS was identified based on diagnostic codes, and 4:1 propensity score matching was conducted. Temporal trends in AS incidence and prevalence were analyzed in terms of sex and age. Kaplan–Meier survival analysis and Cox proportional hazards models were employed for all-cause mortality assessment. **Results**: Among 91,466 eligible patients, 708 (0.77%) had AS. AS incidence and prevalence increased from 8.05 to 35.29 and from 8.05 to 77.43 per 10,000, respectively, from 2009 to 2021, and were higher in women than in men. Age-stratified analysis revealed the greatest burden in patients aged ≥80. AS in the matched cohort (n = 2875) was associated with <10-year survival (13% vs. 24%, *p* < 0.001), with differences evident from age 60 onward. Multivariable analysis revealed AS as an independent mortality predictor (hazard ratio: 1.23, 95% confidence interval: 1.08–1.40, *p* = 0.002). Older age, atrial fibrillation, stroke, and a higher Charlson Comorbidity Index were significant mortality predictors among patients with AS. **Conclusions**: AS burden in dialysis-dependent patients with ESRD is markedly increasing, particularly among women and older adults, and is independently associated with elevated mortality.

## 1. Introduction

Chronic kidney disease (CKD) is a significant and growing public health concern worldwide, with a steadily increasing prevalence and associated mortality [1,2]. Among patients with CKD, those who progress to end-stage renal disease (ESRD) are particularly vulnerable to several complications, including cardiovascular disease (CVD), which remains the leading cause of death in this population [3,4]. Among the various types of CVD, valvular heart disease, particularly aortic stenosis (AS), has exhibited an increasing prevalence, especially in elderly people [5,6]. AS typically develops through degenerative aortic valve calcification, a process that progresses via mechanical stress, chronic inflammation, and metabolic dysregulation [7,8]. Advanced age, bicuspid aortic valve, hypertension, hyperlipidemia, and CKD are major risk factors for AS [5,9].

Multiple studies have revealed a strong association between CKD and AS. AS prevalence increases with advancing CKD stage, and patients with ESRD generally experience more rapid progression of aortic valve calcification, with higher AS prevalence rates reported to be approximately 6.3% in some cohorts [10,11]. Previous studies have reported that AS in ESKD patients develops 10–20 years earlier and progresses more rapidly than in the general population. Dialysis-related factors, including prolonged dialysis duration, hyperphosphatemia, disturbances in mineral metabolism, and systemic inflammation, further accelerate the calcification process in this population [12,13,14]. Moreover, diagnosis may be delayed because typical symptoms can be obscured by comorbid conditions such as anemia, fluid overload, pulmonary hypertension, or coronary artery disease [15]. These challenges highlight the importance of population-based studies to better define the burden and prognostic implications of AS in patients with ESKD. The degree of renal dysfunction is significantly associated with overall survival. Several studies have revealed that more severe kidney impairment is associated with faster AS progression and increased mortality risks or need for aortic valve replacement [11,13]. Furthermore, AS is an independent predictor of cardiovascular mortality in dialysis-dependent patients [10]. These findings indicate that risk factors contributing to AS in patients with ESRD might exhibit varied profile and impact from those in the general population. Identifying the prognostic relevance of AS, as well as dialysis-specific predictors of mortality, in the former group is crucial for improving clinical management and long-term outcomes.

Only a few large-scale studies have investigated the incidence, prevalence, and prognostic implications of AS in Korean ESRD patients despite the clinical importance of AS in ESRD. Further, there is a lack of comprehensive data exploring AS-related mortality and its predictors in this group. Therefore, this study aims to (1) identify the incidence and prevalence of AS in a nationwide cohort of Korean ESRD patients using the Korean National Health Information Database and (2) assess the impact of AS on all-cause mortality and determine associated risk factors that are unique to this high-risk population.

## 2. Materials and Methods

### 2.1. Data Sources

This study used data from the Korean National Health Insurance Service (NHIS), a government-managed, mandatory, single-payer health insurance program that provides universal health coverage for approximately 98% of the Korean population. Further, this database also includes the remaining 2%—medical aid beneficiaries—enabling a comprehensive representation of the entire Korean population.

The NHIS database contains detailed, longitudinal healthcare data, including demographic information, outpatient and inpatient visits, diagnoses, procedures, prescriptions, and health screening results. All diagnoses are coded using the International Classification of Diseases, 10th Revision (ICD-10). Furthermore, procedure and prescription data are recorded based on national insurance billing codes. Reimbursement is tightly linked to claims submission; thus, diagnostic coding in the NHIS database is highly complete and reliable, especially for chronic and serious conditions, including ESRD.

The Institutional Review Board of Seoul National University Hospital (IRB No. EUMC 2023-03-040) approved this study, which was conducted in accordance with the Declaration of Helsinki principles. The requirement for informed consent was waived due to the retrospective study design and use of anonymized administrative data.

### 2.2. Study Population

We identified patients with ESRD who initiated maintenance dialysis between 1 January 2012 and 31 December 2021. Patients with ESRD were defined as those with at least two outpatient or one inpatient claim for ICD-10 code N18 (CKD) or V001 (dialysis registration code in Korea), in combination with at least two claims for dialysis procedure codes: O7010 (hemodialysis), O7020 (peritoneal dialysis), and O7071–O7075 (maintenance dialysis). To ensure that only patients on maintenance dialysis were included, the individuals were required to have received dialysis for at least 90 consecutive days.

Patients who had been diagnosed with AS before index date were excluded. AS was defined as a new diagnosis of ICD-10 code I35.0 (nonrheumatic aortic valve stenosis) after dialysis initiation. The final study cohort included patients who developed AS during dialysis (AS group) and those without any AS diagnosis during the study period (non-AS group).

**Figure 1:**
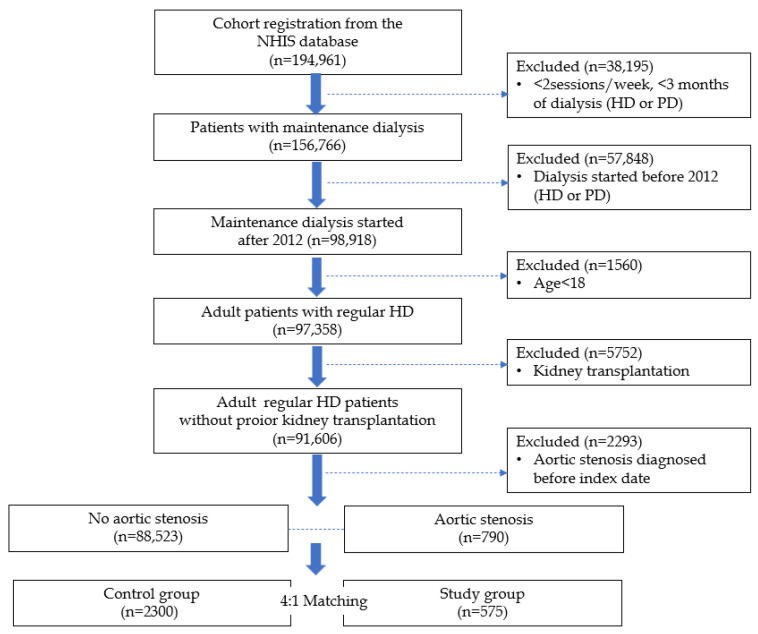
Flow diagram depicting patient selection approach, exclusion criteria, and matching process.

Figure 1 illustrates the step-by-step inclusion process, from the raw dataset to the final matched study population, and summarizes the cohort selection process.

### 2.3. Definitions

AS was defined as the presence of at least two outpatient claims or one inpatient claim coded as I35.0 after dialysis initiation. Comorbid conditions, including diabetes mellitus, hypertension, atrial fibrillation, ischemic heart disease, heart failure, and stroke, were identified using validated ICD-10 codes. The Charlson Comorbidity Index (CCI) was employed to quantify overall disease burden. Date and cause of death were obtained via linkage to the national death registry maintained by NHIS.

### 2.4. Statistical Analysis

Baseline characteristics of the AS and non-AS groups were compared using the chi-square test for categorical variables and Student’s *t*-test for continuous variables. A nearest-neighbor algorithm without replacement was used for 4:1 propensity score matching to reduce confounding bias. Matching variables included age, sex, comorbidities, and CCI score. Matching balance was assessed using standardized mean differences, with values of <0.1 considered acceptable.

We calculated annual incidence and prevalence of AS among patients undergoing dialysis and stratified the results by age group (<50, 50–59, 60–69, 70–79, and ≥80 years) and sex. These trends were visualized in line graphs and described in the Results section with supporting data from Supplementary Tables S1–S4.

Survival analysis was performed using the Kaplan–Meier method, and group differences were assessed using the log-rank test. Cox proportional hazards models were employed to evaluate the association between AS and all-cause mortality. Adjusted models included age, sex, comorbidities, and CCI score. In addition, a subgroup analysis within the AS group was conducted to identify independent mortality predictors. The added prognostic value of clinical variables was assessed using a global chi-square comparison across nested models. All *p*-values of <0.05 indicated statistical significance. SAS Enterprise Guide version 8.2 (SAS Institute Inc., Cary, NC, USA) and R software version 4.0.3 (R Foundation for Statistical Computing, Vienna, Austria) were used for statistical analysis.

## 3. Results

### 3.1. Baseline Characteristics of the Study Population

A total of 91,466 patients met the eligibility criteria. Of them, 708 (0.77%) were diagnosed with AS. Before propensity score matching, the AS group was significantly older (mean age: 75.01 ± 9.77 vs. 64.18 ± 13.94 years, *p* < 0.001) and comprised significantly fewer males (53.5% vs. 60.8%, *p* < 0.001) compared to the non-AS group. The unadjusted hazard ratio (HR) for mortality associated with AS was 2.18 (95% confidence interval [CI]: 1.97–2.42, *p* < 0.001).

To reduce potential confounding, 4:1 propensity score matching was conducted, yielding a final cohort of 2875 patients with ESRD, comprising 575 with AS and 2300 without AS. Baseline characteristics were well balanced between matched groups (Table 1). The mean age was 75.61 ± 10.13 and 71.90 ± 11.79 years in the AS and non-AS groups, respectively (*p* = 0.402), and the proportion of males was comparable in both groups (51.8% vs. 55.4%, *p* = 0.072). Several comorbidities remained more prevalent in the AS group, including atrial fibrillation (23.0% vs. 19.4%, *p* = 0.011), heart failure (63.7% vs. 61.5%, *p* = 0.045), and hyperlipidemia (95.0% vs. 94.3%, *p* = 0.010). All-cause mortality was significantly higher among patients with AS (54.3% vs. 43.7%, *p* < 0.001).

### 3.2. Temporal Trends in the Incidence and Prevalence of AS Among Patients with ESRD According to Sex and Age

**Figure 2:**
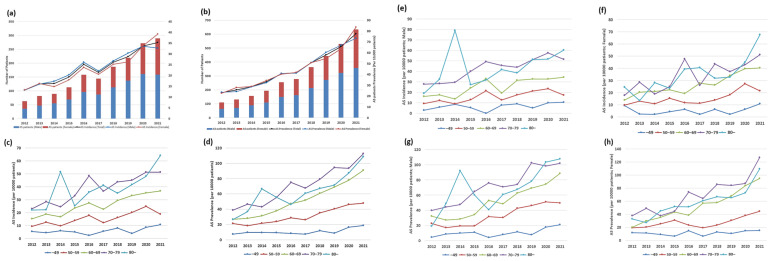
Sex-, age-, and year-specific incidence and prevalence of AS in patients with ESRD. (**a**,**b**) Incidence and prevalence of AS by sex. (**c**,**d**) Incidence and prevalence of AS by age group. (**e**) AS incidence by age group in males. (**f**) AS incidence by age group in females. (**g**) AS prevalence by age group in males. (**h**) AS prevalence by age group in females. AS: aortic stenosis, ESRD: end-stage renal disease.

The incidence and prevalence of AS between 2009 and 2021 markedly increased among patients with ESRD on maintenance dialysis (Figure 2; Supplementary Table S1). The incidence increased from 8.05 to 35.29 per 10,000 patients, whereas the prevalence increased nearly tenfold, from 8.05 to 77.43 per 10,000 patients. The upward trend accelerated after 2015.

Sex-specific analyses revealed a consistently higher burden of AS in women than in men. In 2021, incidence and prevalence of AS in females vs. males were 39.19 vs. 32.60 and 83.51 vs. 73.30 per 10,000 patients, respectively (Figure 2a,b; Supplementary Table S2). Age-stratified trends demonstrated a strong association between age and AS burden, with incidence increasing from 5.22 to 18.89 and from 4.70 to 51.31 per 10,000 patients among those aged 50–59 and 70–79 years, respectively. The highest rates were observed in patients aged ≥80 years, with incidence and prevalence of 64.15 and 108.64 per 10,000 patients, respectively, in 2021 (Figure 2c–h; Supplementary Tables S3 and S4).

### 3.3. AS and Mortality Risk in ESRD

**Figure 3:**
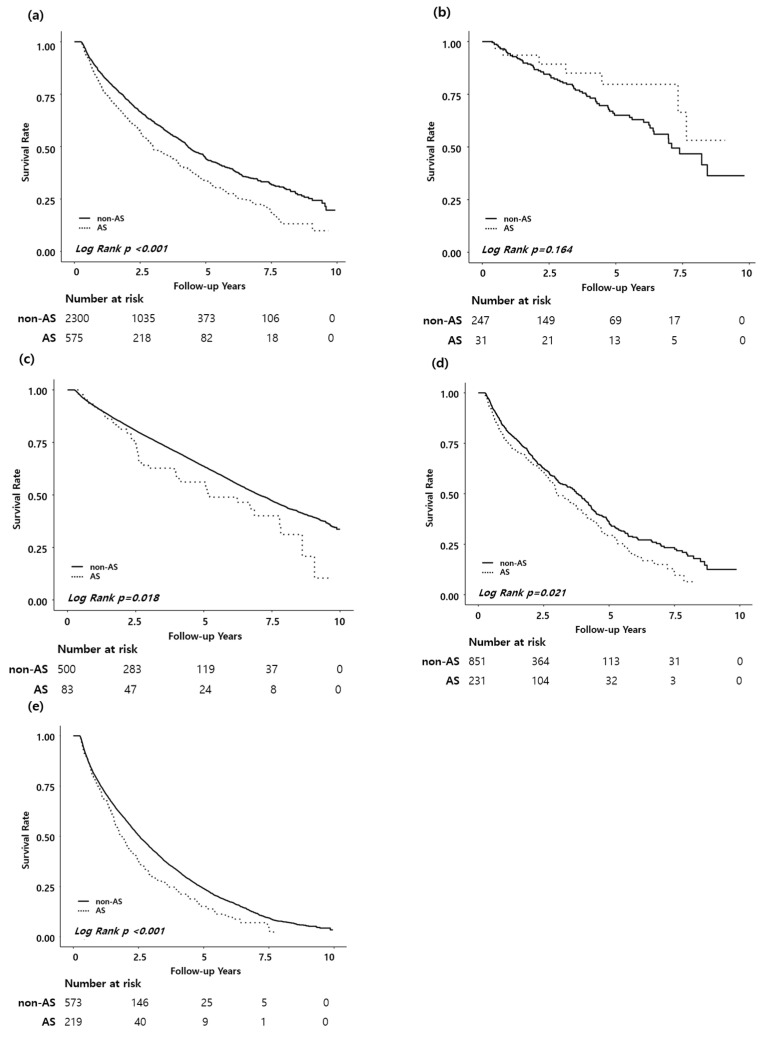
Kaplan–Meier survival curves comparing patients with ESRD with and without AS. (**a**) All ages, (**b**) age ≥ 50 years, (**c**) age ≥ 60 years, (**d**) age ≥ 70 years, (**e**) age ≥ 80 years. AS: aortic stenosis, ESRD: end-stage renal disease.

Kaplan–Meier analysis revealed that AS was significantly associated with reduced overall survival. The 10-year survival rate was 13% in the AS group compared with 24% in the non-AS group (*p* < 0.001). This survival disadvantage became evident from age 60 onward, with statistically significant differences between patients aged ≥60 and ≥70 years. The survival difference was attenuated and not statistically significant in patients aged ≥80 years (Figure 3).

Univariate and multivariate analyses revealed that AS was associated with higher all-cause mortality in Cox regression models. AS remained an independent predictor of death in the fully adjusted model (HR: 1.23, 95% CI: 1.08–1.40, *p* = 0.002). Other independent risk factors included older age (HR for ≥80 vs. <50 years: 8.45, 95% CI: 5.59–12.79, *p* < 0.001), atrial fibrillation (HR: 1.31, 95% CI: 1.16–1.48, *p* < 0.001), stroke (HR: 1.22, 95% CI: 1.07–1.38, *p* = 0.002), and higher CCI score (HR per 1-point increase: 1.06, 95% CI: 1.03–1.10, *p* = 0.001; Table 2).

### 3.4. Predictors of Mortality Among Patients with ESRD and AS

**Figure 4:**
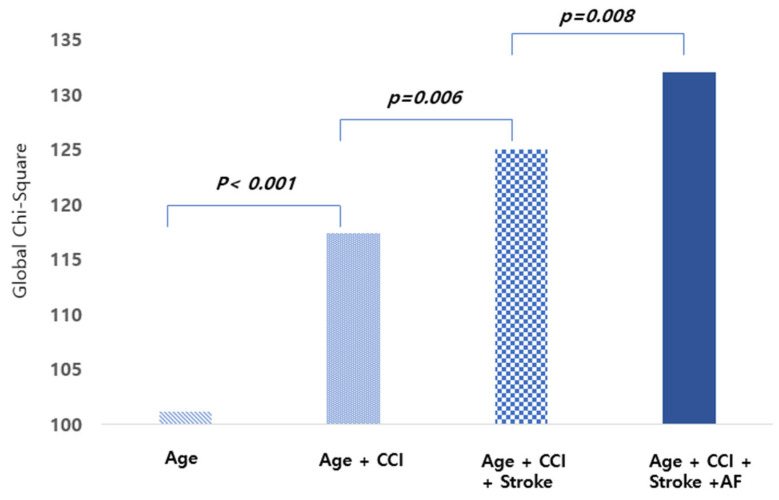
Incremental prognostic value of stroke and atrial fibrillation for mortality in patients with ESRD: Global chi-square analysis. CCI: Charlson comorbidity index; ESRD: end-stage renal disease.

Among patients with ESRD and AS, multivariate analysis identified older age, atrial fibrillation, stroke, and higher CCI as significant predictors of mortality. Compared with patients under 50 years, those aged 70–79 years demonstrated a 5.48-fold higher risk (95% CI: 2.02–14.87, *p* = 0.001), and those aged ≥80 years demonstrated an 8.69-fold higher risk (95% CI: 3.20–23.63, *p* < 0.001). Further, atrial fibrillation (HR: 1.30, 95% CI: 1.05–1.60, *p* = 0.016) and stroke (HR: 1.35, 95% CI: 1.10–1.66, *p* = 0.004) were independently associated with death. Global chi-square analysis supported the incremental prognostic value of these variables (Figure 4; Table 3).

## 4. Discussion

This large, nationwide Korean cohort of patients with dialysis-dependent ESRD revealed a rapidly increasing burden of AS, particularly among women and elderly people. AS incidence between 2009 and 2021 in patients with ESRD increased more than fourfold, whereas prevalence increased nearly tenfold. Importantly, AS was independently associated with a 23% higher risk of all-cause mortality, even after demographic and clinical variable adjustments. These results indicate the growing clinical relevance of AS in the ESRD population and reveal epidemiologic and prognostic patterns that markedly differ from those in the general Korean population.

A recent nationwide Korean study revealed an age-standardized AS incidence and prevalence in the general population of 0.80 and 2.79 per 100,000 persons, respectively, in 2017 [16]. In our study, the corresponding rates in patients with ESRD in 2021 were dramatically higher, 35.29 (incidence) and 77.43 (prevalence) per 10,000 persons, respectively. This striking difference likely reflects accelerated vascular and valvular calcification caused by disordered mineral metabolism, chronic inflammation, and hemodynamic stress unique to the uremic milieu.

In addition, the demographic profile of AS differed across the groups. In the general population, patients with AS were predominantly female (57.3%), had a mean age of 69.9 years, and 80% were aged ≥60 years [16]. Similarly, in our ESRD cohort, patients with AS were older (mean age: 75.6 years) and more often female compared to those without AS. This pattern might indicate survivor bias, delayed diagnosis, or competing mortality in younger patients with ESRD. Notably, the age-specific incidence curve exhibited a steeper increase between the 60s and 70s in patients with ESRD than in the general population, indicating that AS progression may begin earlier in the context of kidney failure. Early progression may remain undiagnosed or overshadowed by other comorbidities until advanced stages. In our cohort, the incidence of AS was high in both sexes, without the female predominance typically reported in the general population [17]. Moreover, the adverse prognostic impact of AS emerged in the 60s, earlier than in the general population, suggesting that ESKD accelerates the clinical consequences of AS. Importantly, this study represents the first nationwide analysis of age- and sex-specific incidence, prevalence, and prognostic implications of AS in an Asian ESKD population. These findings provide novel insights that complement existing Western data and underscore the need for earlier detection and tailored management strategies in this high-risk group. Regarding comorbidity burden, a slightly lower mean CCI was observed in the AS group. This finding may be explained by the scoring structure of the index, which assigns relatively low weight to conditions such as heart failure and atrial fibrillation, while giving greater weight to myocardial infarction, diabetes, and peripheral vascular disease that were more frequent in the non-AS group. In addition, the use of claims data may have contributed to underestimation of comorbidities in older AS patients.

Survival analyses revealed that patients with ESRD and AS demonstrated significantly lower long-term survival than those without AS. Mortality differences became evident from age 60 years—earlier than in the general population, where divergence typically occurs in the 70s [16]—indicating that the prognostic effect of AS manifests sooner in ESRD. The difference was attenuated and not statistically significant in those aged ≥80 years, likely due to competing mortality from other comorbid conditions. Consistent with this, our results showed that the mortality risk associated with AS became markedly higher beginning in patients in their 60s. This finding suggests that regular echocardiographic screening from this age onward may be considered in patients with ESKD to facilitate earlier detection of clinically significant AS. Future prospective studies are warranted to validate the clinical value and feasibility of such an approach.

AS remained an independent predictor of death even after rigorous adjustment using propensity score matching and multivariable Cox regression (adjusted HR: 1.23, 95% CI: 1.14–1.33). This persistent association indicated that the prognostic burden of AS is not simply a reflection of baseline cardiovascular risk in ESRD. Our findings were consistent with population-based studies revealing the adverse prognostic implications of AS across diverse patient groups [16,18,19,20].

Mortality predictors among patients with ESRD and AS in our study differed from those in the general population. Prior nationwide analyses revealed that older age, male sex, and cardiovascular comorbidities, such as heart failure, myocardial infarction, and atrial fibrillation, were significant predictors. In contrast, older age remained the strongest risk factor in our ESRD–AS cohort, but atrial fibrillation was the only cardiovascular condition independently associated with mortality. Stroke history and higher CCI scores were significant predictors, whereas sex was not. In this study, peritoneal dialysis patients comprised only about 2.4% of the total cohort. This small subgroup limits the robustness of mortality analyses according to dialysis modality and precludes drawing firm conclusions about differences between hemodialysis and peritoneal dialysis. Therefore, the observed lack of modality-related mortality differences should be interpreted with caution. These results indicated the multifactorial nature of mortality in patients with ESRD and AS, where both cardiac and systemic comorbidities must be addressed.

The earlier onset and stronger prognostic impact of AS observed in our ESRD cohort indicated that current approaches to detection may be insufficient in this high-risk population. Considering the accelerated progression of valvular calcification in kidney failure, earlier and more frequent echocardiographic screening—particularly beginning in the sixth decade of life—may enable timely identification of clinically significant AS before symptom onset. This is especially relevant for patients with atrial fibrillation or stroke history, who demonstrated the highest mortality risk in our study. Further, multidisciplinary risk stratification that involved nephrologists, cardiologists, and cardiovascular surgeons may be warranted to optimize management decisions, including the timing and appropriateness of aortic valve intervention. The benefit-to-risk balance of surgical or transcatheter valve replacement in ESRD remains uncertain; however, our findings indicated the need for prospective studies to define optimal treatment strategies in this vulnerable population.

This study had some limitations. First, AS cases were based on claims data from the NHIS; thus, we may have missed patients who did not seek medical care or who paid out-of-pocket, potentially causing an underestimation or overestimation of disease burden. Second, the NHIS dataset lacks echocardiographic and procedural information; therefore, AS severity, valve morphology, and hemodynamic parameters could not be evaluated, and interventional outcomes could not be assessed. This limitation precludes detailed risk stratification and interpretation of management outcomes. Accordingly, the optimal evaluation and treatment of AS in ESKD patients should rely on a multidisciplinary pathway involving cardiology, nephrology, and cardiac surgery, and future prospective studies are needed to systematically assess disease severity, progression, and procedural outcomes. Given the inherent limitations of claims data, certain clinical details—such as BMI and specific oncological characteristics—could not be captured, although we incorporated all available comorbidities, including the presence of cancer, for adjustment. Third, although propensity score matching was performed to balance baseline characteristics, some residual differences in comorbidities, such as atrial fibrillation, persisted and may have introduced residual confounding that could have influenced the outcomes. Fourth, procedural data for surgical or transcatheter aortic valve replacement and balloon valvuloplasty were unavailable, which limited our ability to assess the impact of interventions. Finally, most laboratory data originated from health screening records, which generally do not include ESRD-specific markers, precluding full assessment of biochemical contributors to valvular disease.

## 5. Conclusions

This nationwide study reveals a substantial and increasing burden of AS in patients with dialysis-dependent ESRD, with markedly higher incidence, prevalence, and mortality compared with the general population. AS remained an independent mortality predictor after adjustment for comorbidities, particularly among those with atrial fibrillation or stroke. These findings indicate the importance of early detection, risk stratification, and individualized management strategies for AS in patients with ESRD, as well as the need for further research into optimal screening and treatment approaches in this high-risk group.

## Figures and Tables

**Table 1:** Baseline characteristics of patients with ESRD with and without aortic stenosis.

Variables	Total (%)	Non-AS (%)	AS (%)	*p*-Value
Patient number	2875	2300 (80.0)	575 (20.0)	
Male	1572 (54.7)	1274 (55.4)	298 (51.8)	0.072
Age (years)	71.90 ± 11.70	71.90 ± 11.79	75.61 ± 10.13	<0.001
<50	97 (3.4)	83 (3.6)	14 (2.4)	
50–59	278 (9.7)	247 (10.7)	31 (5.4)	
60–69	783 (27.2)	637 (27.7)	146 (25.4)	
70–79	925 (32.2)	760 (33.0)	165 (28.7)	
≥80	792 (27.5)	573 (24.9)	219 (38.1)	
Income				0.004
Low	792 (27.5)	658 (28.6)	134 (23.3)	
Mid Low	357 (12.4)	278 (12.1)	79 (13.7)	
Mid High	505 (17.6)	388 (16.9)	117 (20.4)	
High	1221 (42.5)	976 (42.4)	245 (42.6)	
Dialysis				0.026
Hemodialysis	2806 (97.6)	2243 (97.5)	563 (97.9)	
Peritoneal dialysis	69 (2.4)	57 (2.5)	12 (2.1)	
Atrial fibrillation	671 (23.3)	543 (23.6)	132 (23.0)	0.011
Cancer	720 (25.0)	566 (24.6)	154 (26.8)	0.050
Diabetes mellitus	2521 (87.7)	2029 (88.2)	492 (85.6)	0.159
Heart failure	1801 (62.6)	1416 (61.6)	385 (67.0)	0.045
Hyperlipidemia	2725 (94.8)	2179 (94.7)	546 (95.0)	0.010
Hypertension	2844 (98.9)	2270 (98.7)	574 (99.8)	0.132
Myocardial infarction	398 (13.8)	331 (14.4)	67 (11.7)	0.081
Peripheral vascular disease	1330 (46.3)	1074 (46.7)	256 (44.5)	0.044
Stroke	941 (32.7)	672 (29.2)	172 (29.9)	0.076
Charlson Comorbidity Index	7.77 ± 2.62	7.80 ± 2.72	7.63 ± 2.63	0.065
Death	1318 (45.8)	1006 (43.7)	312 (54.3)	<0.001

AS: aortic stenosis; ESRD: end-stage renal disease.

**Table 2:** Mortality predictors in patients with ESRD: Multivariate Cox regression including aortic stenosis.

	Univariate Analysis			Multivariate Analysis		
Variables	HR	95% CI	*p*-Value	HR	95% CI	*p*-Value
Aortic stenosis	1.41	1.24–1.60	<0.001	1.23	1.08–1.40	0.002
Male	1.05	0.94–1.17	0.383			
Age (year)	1.06	1.05–1.07	<0.001	1.06	1.05–1.06	<0.001
<50	ref					
50–59	1.91	1.21–3.01	0.006			
60–69	2.46	1.61–3.75	<0.001			
70–79	5.18	3.44–7.80	<0.001			
≥80	8.45	5.59–12.79	<0.001			
Income						
Low	ref					
Mid Low	0.82	0.67–1.00	0.050			
Mid High	0.92	0.78–1.09	0.344			
High	1.09	0.95–1.24	0.209			
PD	1.17	0.84–1.62	0.365			
Comorbidities						
AF	1.55	1.38–1.75	<0.001	1.31	1.16–1.48	<0.001
HTN	1.50	0.85–2.64	0.163			
DM	1.45	1.22–1.71	<0.001	1.08	0.89–1.31	0.436
HLD	1.27	1.01–1.61	0.044	0.78	0.61–0.99	0.045
MI	1.34	1.15–1.56	<0.001	1.17	1.00–1.37	0.056
HF	1.39	1.25–1.56	<0.001	1.02	0.90–1.16	0.724
PVD	1.14	1.02–1.27	0.023	0.87	0.77–0.98	0.023
Stroke	1.43	1.28–1.60	<0.001	1.22	1.07–1.38	0.002
Cancer	1.35	1.19–1.53	<0.001	1.02	0.87–1.20	0.786
CCI	1.11	1.09–1.13	<0.001	1.06	1.03–1.10	0.001

ESRD: end-stage renal disease; HR: hazard ratio; CI: confidence interval; PD: peritoneal dialysis; AF: atrial fibrillation; HTN: hypertension; DM: diabetes mellitus; HLD: hyperlipidemia; MI: myocardial infarction; HF: heart failure; PVD: peripheral vascular disease; CCI: Charlson Comorbidity Index; AS: aortic stenosis.

**Table 3:** Predictors of mortality in patients with ESRD and AS.

	Univariate Analysis			Multivariate Analysis		
Variable	HR	95% CI	*p*-Value	HR	95% CI	*p*-Value
Male	1.23	1.01–1.49	0.038	1.01	0.83–1.23	0.932
Age (year)	1.06	1.05–1.08	<0.001	1.06	1.05–1.07	<0.001
<50	ref					
50–59	1.43	0.47–4.40	0.530			
60–69	2.69	0.97–7.44	0.057			
70–79	5.48	2.02–14.87	0.001			
≥80	8.69	3.20–23.63	<0.001			
Income						
Low	ref					
Mid Low	1.00	0.71–1.41	1.000			
Mid High	1.08	0.80–1.46	0.625			
High	1.24	0.97–1.58	0.093			
PD	1.43	0.80–2.53	0.227			
AF	1.43	1.16–1.76	0.001	1.30	1.05–1.60	0.016
HTN	0.87	0.12–6.17	0.886			
DM	1.28	0.99–1.67	0.064			
HLD	1.19	0.77–1.82	0.436			
MI	1.31	0.99–1.71	0.055			
HF	1.63	1.32–2.01	<0.001	1.14	0.91–1.43	0.266
PVD	1.28	1.05–1.56	0.014			
Stroke	1.58	1.29–1.93	<0.001	1.35	1.10–1.66	0.004
Cancer	1.18	0.94–1.47	0.155			
CCI	1.13	1.09–1.17	<0.001	1.07	1.03–1.12	0.001

Abbreviation: ESRD: end-stage renal disease; HR: hazard ratio; CI: confidence interval; PD: peritoneal dialysis; AF: atrial fibrillation; HTN: hypertension; DM: diabetes mellitus; HLD: hyperlipidemia; MI: myocardial infarction; HF: heart failure; PVD: peripheral vascular disease; CCI: Charlson Comorbidity Index; AS: aortic stenosis.

## Data Availability

The authors are restricted from sharing the data underlying this study because the Korean National Health Insurance Service (NHIS) owns the data. Researchers can request access to the NHIS website (https://nhiss.nhis.or.kr, accessed on 27 March 2025). Details of this process and the provisional guide are available at (http://nhiss.nhis.or.kr/bd/ab/bdaba000eng.do, accessed on 27 March 2025).

## Supplementary Materials

The following supporting information can be downloaded at: https://www.mdpi.com/article/10.3390/jcm14196921/s1, Supplementary Table S1: Annual incidence rate and prevalence (%) of AS in ESRD patients in Korea (2009–2021), Supplementary Table S2. Sex-specific incidence rate and prevalence (%) of AS in ESRD patients in Korea (2009–2021). Supplementary Table S3. Age-specific incidence rate and prevalence (%) of AS in ESRD patients in Korea (2009–2021). Supplementary Table S4. Age- and sex-specific incidence rate and prevalence (%) of AS in ESRD patients in Korea (2009–2021).

## Abbreviations

The following abbreviations are used in this manuscript:

CKD	Chronic kidney disease
ESRD	End-stage renal disease
CVD	Cardiovascular disease
AS	Aortic stenosis
NHIS	Korean National Health Insurance Service
ICD-10	International Classification of Diseases, 10th Revision
CCI	Charlson Comorbidity Index
HR	Hazard ratio
CI	Confidence interval
PD	Peritoneal dialysis
AF	Atrial fibrillation
HTN	Hypertension
DM	Diabetes mellitus
HLD	Hyperlipidemia
MI	Myocardial infarction
HF	Heart failure
PVD	Peripheral vascular disease
