# Supplementary material for: Aortic Stenosis in End-Stage Renal Disease: Incidence, Prevalence, and Mortality in a National Korean Cohort
Source: J Clin Med. 2025 Sep 30;14(19):6921. doi: 10.3390/jcm14196921 (PMC12525193; doi:10.3390/jcm14196921)
Supplement: Supplementary file 1 [file jcm-14-06921-s001.zip › jcm-3862694-supplementary.pdf]

Article

Aortic Stenosis in End-Stage Renal Disease: Incidence, Prevalence, and Mortality in a National Korean Cohort

Minjeong Kim[1,2], Min Ho Kim, Hyangkyoung Kim[1]

Supplementary Table S1. Annual incidence rate and prevalence (%) of AS in ESRD patients in Korea (2009–2021).

Year	Incidence rate (per 10,000 PY)	Prevalence (%)
**2012**	13.26	0.23
**2013**	16.07	0.26
**2014**	16.29	0.29
**2015**	19.3	0.33
**2016**	25.18	0.41
**2017**	21.46	0.41
**2018**	26.14	0.51
**2019**	28.73	0.58
**2020**	33.66	0.66
**2021**	35.31	0.77

ESRD: end-stage renal disease; AS: aortic stenosis.

Supplement Table S2. Sex-specific incidence rate and prevalence (%) of AS in ESRD patients in Korea (2009–2021).

Year	Male Incidence rate (per 10,000 PY)	Male Prevalence (%)	Female Incidence rate (per 10,000 PY)	Female Prevalence (%)
**2012**	13.23	0.24	13.29	0.23
**2013**	15.94	0.24	16.24	0.28
**2014**	17.36	0.28	14.82	0.29
**2015**	20.25	0.32	17.96	0.34
**2016**	26.18	0.41	23.75	0.40
**2017**	22.07	0.41	20.57	0.42
**2018**	26.80	0.51	25.18	0.51
**2019**	30.37	0.60	26.37	0.55
**2020**	33.59	0.67	33.77	0.64
**2021**	32.64	0.73	39.19	0.84

ESRD: end-stage renal disease; AS: aortic stenosis.

Supplement Table S3. Age-specific incidence rate and prevalence (%) of AS in ESRD patients in Korea (2009–2021).

**Incidence/10,000**										
**Age Group**	**2012**	**2013**	**2014**	**2015**	**2016**	**2017**	**2018**	**2019**	**2020**	**2021**
**<50**	5.44	4.39	5.99	5.01	2.44	5.64	7.99	3.95	8.62	10.58
**50–59**	9.42	12.51	9.72	13.88	17.77	12.13	16.19	20.15	24.81	18.87
**60–69**	15.11	18.74	16.65	23.43	27.41	22.60	29.32	33.16	35.25	36.69
**70–79**	22.99	28.43	24.52	32.95	48.50	36.60	43.76	45.07	51.17	51.31
**≥80**	21.98	22.09	51.52	25.26	35.75	41.07	35.10	41.56	48.13	64.15

Prevalence/10,000										
**<50**	7.25	9.67	9.41	9.18	8.14	7.25	12.00	8.68	16.45	18.71
**50–59**	21.20	18.40	21.53	23.79	28.56	26.09	35.27	40.30	46.31	47.73
**60–69**	26.86	28.11	31.13	37.75	47.07	51.80	60.69	68.69	77.91	90.90
**70–79**	38.67	46.44	42.90	55.20	75.03	67.67	79.27	94.29	93.44	112.67
**≥80**	26.37	36.82	66.67	55.57	46.27	60.71	67.16	71.43	87.31	108.64

ESRD: end-stage renal disease; AS: aortic stenosis.

Supplement Table S4. Age- and sex-specific incidence rate and prevalence (%) of AS in ESRD patients in Korea (2009–2021).

**Incidence/10,000**											
**Age Group**	**Sex**	**2012**	**2013**	**2014**	**2015**	**2016**	**2017**	**2018**	**2019**	**2020**	**2021**
**<50**	**Male**	5.44	4.39	5.99	5.01	2.44	5.64	7.99	3.95	8.62	10.58
**<50**	**Female**	7.25	9.67	9.41	9.18	8.14	7.25	12.00	8.68	16.45	18.71
**50–59**	**Male**	9.42	12.51	9.72	13.88	17.77	12.13	16.19	20.15	24.81	18.87
**50–59**	**Female**	21.20	18.40	21.53	23.79	28.56	26.09	35.27	40.30	46.31	47.73
**60–69**	**Male**	15.11	18.74	16.65	23.43	27.41	22.60	29.32	33.16	35.25	36.69
**60–69**	**Female**	26.86	28.11	31.13	37.75	47.07	51.80	60.69	68.69	77.91	90.90
**70–79**	**Male**	22.99	28.43	24.52	32.95	48.50	36.60	43.76	45.07	51.17	51.31
**70–79**	**Female**	38.67	46.44	42.90	55.20	75.03	67.67	79.27	94.29	93.44	112.67
**≥80**	**Male**	21.98	22.09	51.52	25.26	35.75	41.07	35.10	41.56	48.13	64.15
**≥80**	**Female**	26.37	36.82	66.67	55.57	46.27	60.71	67.16	71.43	87.31	108.64
**Prevalence/10,000**											
**<50**	**Male**	3.76	5.01	4.88	4.75	4.21	3.73	6.21	4.49	8.52	9.69
**<50**	**Female**	3.49	4.66	4.53	4.43	3.99	3.52	5.79	4.19	7.93	9.02
**50–59**	**Male**	10.99	9.53	11.16	12.33	14.80	13.52	18.27	20.84	23.92	24.73
**50–59**	**Female**	10.21	8.87	10.37	11.46	13.75	12.57	16.99	19.46	22.39	23.00
**60–69**	**Male**	13.90	14.62	16.17	19.60	24.45	26.83	31.46	35.56	40.31	46.99
**60–69**	**Female**	12.94	13.61	15.06	18.25	22.73	24.97	29.23	33.13	37.60	43.91
**70–79**	**Male**	20.09	24.03	22.19	28.53	38.75	34.96	40.99	48.79	48.42	58.32
**70–79**	**Female**	18.58	22.23	20.51	26.37	35.78	32.71	38.28	45.50	45.02	54.35
**≥80**	**Male**	13.65	19.73	35.54	29.01	24.00	31.46	34.77	36.99	45.23	56.21
**≥80**	**Female**	12.77	18.44	33.11	27.02	22.35	29.29	32.39	34.47	42.08	52.43

ESRD: end-stage renal disease; AS: aortic stenosis.

Disclaimer/Publisher's Note: The statements, opinions and data contained in all publications are solely those of the individual author(s) and contributor(s) and not of MDPI and/or the editor(s). MDPI and/or the editor(s) disclaim responsibility for any injury to people or property resulting from any ideas, methods, instructions or products referred to in the content.
